# Predictors of Self-medication in Iran: A Notional Survey Study

**DOI:** 10.22037/ijpr.2020.113601.14394

**Published:** 2021

**Authors:** Sajad Vahedi, Faride Sadat Jalali, Mohsen Bayati, Sajad Delavari

**Affiliations:** a *Department of Health Service Management, School of Health, Ahvaz Jundishapur University of Medical Sciences, Ahvaz, Iran. *; b *Health Human Resources Research Center, School of Management and Information Sciences, Shiraz University of Medical Sciences, Shiraz, Iran.*

**Keywords:** Self-medication, Health behavior, Drug-seeking behavior, Consumer behavior, Health insurance, Patient Acceptance of Health Care, Health Care Seeking Behavior

## Abstract

While logical use of medicine is a priority in all health systems, people do self-medication- mainly using Nonprescription Drugs or Over the Counter (OTC) drugs- for different reasons. Self-medication is rising in many developing countries that could increase healthcare expenditure. The present study aimed to find the self-medication rate and predisposing, enabling, and need factors affecting it based on the Anderson behavioral model in the Iranian population. The present study uses 22470 households’ data acquired from Iranian utilization of healthcare survey at the national level (2016). Due to the study objective, the data of 13005 people who were over 15 years old and had outpatient healthcare needs two weeks before the survey. The survey included a binary question about self-medication, which is considered a dependent variable. Age, gender, marital status, literacy, job status, socio-economic status, location, basic health insurance, complementary health insurance, and need for health services were considered as independent variables. Data were analyzed using logistic regression. The self-medication rate was calculated at 26.3% that was different among different subgroups of the population. According to the model estimates, married (OR = 0.80, CI = 0.71-0.91) and housekeepers (OR = 0.79, CI = 0.67-0.93) had significantly lower self-medication. Moreover, the urban population (OR = 1.29, CI = 1.17-1.43), people without basic (OR = 1.32, CI = 1.10-1.58), and supplementary (OR = 1.18, CI = 1.04-1.35) health insurance and also people who had two or higher number of outpatient healthcare needs had significantly more self-medication (OR = 2.96, CI = 2.67-3.29). It can be concluded that need, enabling, and predisposing factors are respectively the main determinants of self-medication behavior. From a policy point of view, increasing effective health insurance coverage with a focus on people who have more health care needs can be helpful.

## Introduction

A healthy human being is at the heart of sustainable development in any society, and in this regard, the role of medication is crucial. Medicinehas always been one of the ways of fighting diseases (1). It can be said that the emergence of pharmaceuticals dates back to the history of human existence. Today, medicine is one of the most expensive inputs in the health system. A great deal of money is spent on it in all countries (10% to 20% of health expenditure in developed countries and 20% to 40% in developing ones) (2, 3). Research results show that more than 50% of prescriptions, distributions, or sales of medicines in the world are inappropriate (4).

With significant advances in various sciences, more and more people are getting access to different medicines,so that easy access to medicineshas led to improper use of medicines by people (5). Proper and rational use of medicines has a significant impact on controlling the costs of health sector and needs to be addressed by the community and policymakers. According to the World Health Organization’s (WHO) estimates, over half of all drugs in the world are inappropriately prescribed or sold, and 50% of patients fail to use them properly (6). Some studies showed that about 30% of liver and kidney disorders were due to improper and inappropriate drug use, and 3% of all the patients admitted to the US hospitals were drug abusers (7).While medication is an important component of any disease treatment and 75% of them are treated by drugs, irrational use of them not only fails to treat diseases but also causes long-term drug side effects (8). Unfortunately, the public only sees the safe and healing aspect of taking drugs, while in medical literature, medicine is thought of like a double-edged razor, with one edge facing pathogens and the other attackinghuman lives due to the lack of knowledge of using them properly (9).

In addition to taking prescribed medications by physicians, many people nowadays go directly to pharmacies and buy and take various medications based on their own diagnoses, which are often wrong, to prevent or treat diseases or even strengthen their bodies (10). As one of the most common forms of inappropriate medication use, self-medication is defined by the WHO as preparing and taking medicines to treat self-recognized ailments or symptoms without a physician’s opinion. Self- medication involves the use of herbal or synthetic drugs (11, 12). It has various forms, such as taking medications without a physician’s prescription for treatment in situations similar to the previous courses of illnesses, using medications available at home, and failing to heed the physician’s recommendations (13). Arbitrary drug use has now led to incidences, such asincreased bacterial resistance, lack of optimal treatments, unwanted and even deliberate poisonings, side effects and adverse drug reactions, disruption in the drug market, waste of resources, and increased per capita drug use in the community (14). 

Arbitrary drug use as the first choice of patients is common in many communities worldwide and rapidly increases (15, 16). The prevalence of self-medication varies in different regions of the world, depending on different cultural, political, and economic factors. For example, the prevalence of self-medication is about 68% in European countries (17), 77% in the US, 31% in India (7), 21.5% in rural areas of Portugal, and 14.9% among Brazilian adults (18). The rate of medicineuse in Iran has been increasing in recent decades. The increasing rate is not in line with the epidemiological conditions of diseasesand the population growth, as the main variables whichcan be related to self- medication (19). The rate of self-medication in Iran is higher than the mean world rate (13). However, various local studies reported a 36% to 83% prevalence of self-medication among Iranians (20).

Arbitrary drug use is a serious threat to the health of the community, and the resolution of this problem requires proper education and information delivery to the general public (21). It is evident that irrational and arbitrary drug use is misconduct among households and needs to be identified and investigated (16). Drug use in Iran lacks the right pattern, and efforts to correct it have not been successful, and the drug system is still facing arbitrary drug use. Hence, it is essential to identify the causes of self-medication and provide a solution to reduce it. Although various studies were conducted on self- medication in different regions and among different social groups in Iran, none of them addressed the issue comprehensively and at a national level. Therefore, the present study aimed to investigate the factors affecting arbitrary drug use in Iran.

## Experimental

*Study setting and source of data*

A cross-sectional design was used to determine socio-economic predictors of self-medication in Iran. Required data in this study is derived from the nationally representative survey conducted in the collaboration of the National Institute of Health Research and Statistical Center of Iran over a twenty-day period in 2016 (3–22 January) through three-stage cluster sampling design. Entitled Iranian Utilization of Healthcare services (IRUHS), the primary goal of this survey was preparation of relevant data about utilization status of healthcare services and determining potential factors that modify it in Iran. In this survey, 22470 households from the whole country were chosen. Two questionnaires entitled Household Questionnaire (to collect household-level and individual characteristics and healthcare needs) and Individual Questionnaire (to collect detailed information about utilization of healthcare services) were used in this study and were completed through face-to-face interviews. In the first step, 76674 individuals responded to the Household Questionnaire (response rate: 96.6%). Finally, in the second step, 20313 individuals that had reported healthcare need in the former research, were selected. Because of recalling bias problem, this study only focused on 15-year-oldor higher individuals that had outpatient healthcare needs (N = 13005).

*Definition of variables and statistical analysis*

In this study, the medication status of those that reported outpatient healthcare needs to be reviewed to define self-medication. The answer of those that used outpatient healthcare services to this question “Have you taken any medicine through self-referral to a pharmacy without prescription in the last two weeks?”, and answer of those that avoid seeking outpatient healthcare services to this question “Have you use any medicine from former medicine in your home in the last two weeks?” was used to measure self-medication. Hence, self-medication defined as a medication of those that used any medication from a pharmacy without prescription and those that avoid seeking outpatient healthcare services and utilized from former medicines in their homes in the last two weeks.

Potential predictors of self-medication were studied through Andersen’s Behavioral Model of Health Services Use. This model assumes that health-seeking behavior (22), such as self-medication (23) is a function of predisposing, enabling, and need factors. Demographic characteristics such as age, gender, marital status, and social structure such as occupation, education, and ethnicity in this model are categorized as predisposing factors. Enabling factors include material resources such as income or economic status, possession of health insurance, and also the distance from healthcare providers. Finally, health status measures such as self-rated health, health-related quality of life, or chronic conditions could be used as a need factor.

Based on Anderson behavioral model, predisposing factors in this study were included gender (male or female), age groups (15-29, 30–44, 45-59, 60-74 and 75+), marital status (married or unmarried), education (illiterate, primary, secondary, high school diplomaand higher education) and occupational status (employed, unemployed, having income without employment, student and housekeeper). Area of residence (urban or rural), wealth quintals (Q1, Q2, Q3, Q4, and Q5), and possession of health insurance (basic and supplementary) were considered as enabling factors. Asset data such as having a separate kitchen, central heating, telephone usage, computer, Internet access at home, owning a motorcycle, car and whether the person owned the house or not were used in the principal component analysis, as a statistical scheme when no quantitative variable, such as income or expenditure exists, to create wealth index (24). Finally, the number of outpatient healthcare needs (one or two and higher) was used as a need factor in this study. Hence, the answer of those that participated to the survey to this question, “Did you have any outpatient healthcare need in the last two weeks?” was used to define need variable. 

Bivariate analyses using chi-square tests were performed for each predictor variable. A step-wised logistic regression model, based on Andersen’s conceptual, was employed to identify predictor variables associated with self-medication. Hence, three consecutive logistic models were estimated. In the first model, independent variables only included predisposing factors. In the second model, enabling factors were added to predisposing factors. And finally,in the third model, the number of outpatient healthcare needs as need factors were included in the analysis. To represent the Iranian population, “weighting procedures” were used in all estimated logistic regression models. All analyses were performed using STATA SE version 12 (Stata Corporation, College Station, Texas, USA). Statistical significance was considered at *p*-value ≤ 0.05, and Odd ratios (OR) with 95% confidence intervals (95% CI) were reported for each variable.

## Results

About 60, 75, and 47 percent of the whole sample were female, married, and housekeeper, respectively. Other characteristics of the surveyed population are shown in the second column of Table 1.

We found that 3,416 of 13005 respondents (who had outpatient healthcare needs in the two weeks preceding the survey) had self-medication, so the rate of self-medication was 26.27%. This rate was different across different subgroups. People with two and higher outpatient healthcare needs (44.99%), more than 75 years old (31.81%), and without basic health insurance (31.19%) had the highest rate of self-medication. Conversely, people with only one healthcare need (21.62%), with supplementary health insurance (22.61%) and richest ones (22.84%) had the lowest rate of self-medication. The bivariate analysis also showed that there is a significant (*p *< 0.01) difference in self-medicationbased on age, marital status, education level, employment status, area of residence, economic status, basic health insurance, supplementary health insurance, and number of outpatient healthcare needs (Table 1).

Logistic regression estimates of Andersen’s Behavioral Model for predisposing, enabling, and need factors affecting self-medication are presented in Table 2. 

According to the first model,which includes only predisposing factors, married people had significantly lower self-medication (OR = 0.79, CI = 0.70-0.89). People with lower education levels had significantly higher self-medication; for example, illiterate persons had significantly higher self-medication rate (OR = 1.73, CI = 1.44-2.07) than the ones who have higher education. Moreover, people with income without a job (OR = 0.80, CI = 0.67-0.95) and housekeepers (OR = 0.77, CI = 0.65-0.91) had significantly lower self-medication than employed people.

In the second model, which assesses predisposing and enabling factors, people with higher ages had significantly more self-medication than the reference age group (15-29), married people, and housekeepers had significantly lower self-medication.The urban population (OR = 1.25, CI = 1.13-1.38) had more self-medication than the rural. People in lower socio-economic status had more self-medication compared to the richestsocio-economic status group. Moreover, people without basic (OR = 1.24, CI = 1.03-1.48) and Supplementary (OR = 1.22, CI = 1.07-1.39) health insurance had significantly more self-medication.

According to the third model, which includes all predisposing, enabling, and need factors, similar to other models, married and housekeeper populations had significantly lower and urban population, people without primary and supplementary health insurance had the substantially higher self-medication.Moreover, people who had two or higher outpatient healthcare needs had significantly more self-medication (OR = 2.96, CI = 2.67-3.29).

## Discussion

Self-medication is widely observed and expanding worldwide, especially in developing countries (25). Although medicine use is one of the critical links in the treatment chain for many diseases, overuse and arbitrary use of medicines can be a sinificant problem in the health system. It could have side effects and risks for peopleas well as high costs imposed on the national pharmaceutical budget, insurance companies, and people (26). Therefore, the present study aimed to investigate the factors affecting arbitrary drug use in Iran in 2016.

According to the results of this study, the rate of self-medication among Iranianswas 26.27% in 2016. However, studies carried out in Iran, and other countries reported differentself-medication rates.

This rate was reported 87.3% among medical and dentistry student in south India (17), 57% in Nigeria (15), 81.77% among the general population of India (27), 80.5% among the middle-aged Brazilian population, 22% in Spain (28), 15% in France (29), 50.1% among non-medical university students in Karachi (30), 21.5% in rural areas of Portugal (31), 42.5% among Jordanians (32), 27.1% among Serbian adults (33), 35.9% in Meket (34) and 22.5% for antibiotics in the Algarve region (35). Iranian Studies also report different rates for self-medication. Self-medication prevalence was reported 72% among Iranian medical students (36), 76% among Iranian women (37), 84.5% in Birjand city (25), 53.6% in the southeastern part of Iran (38), 78.7% in the southern areas of Iran (39), 35.4% in the western part of Iran (26), 41.1% in Ardabil city (21), 32.8% in Tehran City (40), 91% among students and patients with migraine in Kerman city (41) and 89.6% in health sciences students in Kermanshah city (42).

One of the reasons for the differencesinself-medication investigation resultsin the present study andthe most similar ones is the shorter recall period (two weeks interval). The recall period was six months in the study by Niroomand *et al.* (36), three months by Bekele *et al.* (2015) in Ethiopia (43), two months by Olumide *et al.*, and six months by Filipe *et al*. (2016) in France (15, 29). The differences in the populations under study and the sample sizes might also lead to different results. Naik *et al.* (2019), Karimi *et al.* (2019), and Sedighi *et al. *studied 300 medical and dental students (17), 360 Iranian women (13), and 210 school and university students with migraine headaches in Kerman (41), respectively. Another reason for obtaining different results is how self-medicationwas defined and measured. According to studies conducted in other countries, developed nationshadlower self-medication rates than less developed countries. Besides, a look at the statistics shows that although self-medication is widespread worldwide, it is also prevalent in Iran and should be considered as one of the major challenges in the health sector of the country.

In the following, the factors influencing self-medication are investigated using Anderson’sbehavioral model.

Self-medication frequency based on sex, age, marital status, education, and employment showed a higher rate among males (27.02%), age group ≥75 years (31.81%), single (29.37%), illiterate (31/10%), and unemployed people (28/09%).

According to the model’s estimates, there was no significant relationship between sex and self-medication. Regarding age, there was a significant relationship in models two and three, but none was found in model one. In all three models, a significant relationship was observed between marital status and self-medication, and this behavior was less common among married people. About education, there was a significant relationship in model one but no significant relationship was found in the other two models. In all three models, the rate of self-medication was lower in housewives than in other groups.

About demographic (predisposing) variables, studies revealed different findings. For example, some studies showed that men self-medicate more than women (25), while others found that women practice more self-medication (44-46). On the other hand, some studies showed that there is no difference between men and women (23, 36, 37 and 43). While some studies found married people do more self-medication (38) more other studies found single people self-medicate more (25, 44, 47 and 48) which are in line with our findings. On the other hand, some studies found no difference between married and single individuals (36, 37). Some studies revealed age has a positive correlation with self-medication (13, 44).On the other hand, some studies found no correlation (37). According to most studies, education level is another demographic variable that hasa negative relation with self-medication (13, 25, 38 and 49). Several studies revealed contradictory findings of education level (44, 50 and 51). Lei (2018) found no relation between self-medication and education level (23). Having a job has no definite relation to self-medication. Many studies showed no association between self-medication and job type (36, 37), while some studies found some working groups such as housekeeper women (25) and self-employed (50) practice more self-medication than others. 

Predisposing factors such as age, marital status, education, and occupation might have important effects on people’s attitudes and beliefs about medication use. For instance, the significant relationship between education and self-medication in this study wasmost likely becausehigher education levels would increase the awareness of the harmful effects of self-medication, particularly the drugs that require a doctor’s prescription, and this can in turn prevent consumption.

The reason for low self-medication in the lower age groups was that they considered diseases more serious and they were more vulnerable to self-treatment. On the other hand, a greater need for health services and, consequently, more drug use could be some causes of increased self-medication in older age groups.

It also appears that more emotional attention and support from married people than single ones, and encouraging and forcing one’s spouse to refer a physician when the disease occurs may result in lower self-medicationrates among married people.Tirgar et al.stated that the spouses’ persuasionto going to the physicians could be a reason for higher self-medication in married people (52). 

In the present study, the prevalence of self-medication was higher inthe first income quintile (poorest households) (28.29%), and those who lacked basic (31.19%) and supplementary (27.12%) health insurance coverage.

The regression estimates about enabling factors showed that urban residents had significantly more self-medication than rural ones. According to the second model, self-medication was lower in the lower socio-economic groups. It was considerably higher in the groups without basic and supplementaryhealth insurance than those with insurance coverage. 

Rezaei *et al.* (2015) showed that the self-medication rates was higher in people without insurance coverage (16). Two other studies indicated that people with rural insurance had higher rates of self-medication (13, 53). In the study by Tahergourabi *et al.*, self-medication was higher in poorer households and those without basic and supplementary insurance (25). In a study in turkey insured people had less use of non-prescribed medicine (46). The highest rates of self-medication in the studies by Selvarj *et al.* (2014) in India and Cindy *et al.* (1994) in Hong Kong were found among the fourth income quintile (51) and the third social quintile (54), respectively.

It seems that sinceinsurance companies pay for the doctors’ visits and medicines of the people with basic and supplementary insurance coverage, they go to doctors at theevent of disease and take medication as prescribed. Hence, the rate of their self-medication is lower. Birghadr *et al.* (2012) reported that self-medication was higher among people who lacked basic and supplementary insurance coverage (55) due to the use of home-available medicines or arbitrary purchase of medicines from pharmacies (in order not to pay additional costs for visits). In other studies, the highest prevalence of self-medication was among people who were not covered by any insurance (38, 53).

Some studies found that the reasons for the higher rate of self-medication in urban areas. The reasons werethe low quality of and satisfaction from health services in cities, crowded cities, and lack of time of urban citizens, which in turn would lead to drug storage at home and arbitrary drug use (56, 57).

The prevalence of self-medication in people who needed outpatient services for more than twice was much higher (44.99%) than those who needed them once. Furthermore, the third model indicated that self-medication was higher in people with more than one need for health services. Not having enough time for frequent visits to physicians, high costs of doctor visits and lack of financial capacity, and lacking adequate insurance coverage might be the other causes of higher self-medication in the groups with higher treatment needs, as mentioned in some studies (13, 21 and 27). In addition, several studies revealedthat some of the most common causes of increased self-medication among patients with higher ailment frequencies were previous drug use experiences, obtaining sufficient information on drugs and diseases, and similarity of their current diseases with previous ones (7, 54 and 58).

This research has several strengths compared to other similar studies carried out in Iran. It was conducted at a national level with a remarkable sample size and a rigorous sampling design. Doing an analysis based on a strong theoretical basis (Andersen’s Behavioral Model of Health Services Use) was the strength of the present study. However, a main limitation was that our analysis was based on self-reported data that could increase recall bias. Another limitation was the time horizon of study.In other words, the current study was based on a cross sectional survey in a year (2016) which cannot assess self-medication behavior of people across time.

**Table 1:** Characteristics of the whole-sample and population with and without self-medication in Iran 2016

**Overall **			**Whole-sample**	**Population with self-medication**	**Population without self-medication**		***p*** **-value**
			**N = 13005**	**3,416 (26.27%)**	**9,589 (73.73%)**		
**Predisposing factors**							
Sex	Male		5,210 (40.06‌%)	1,408 (27.02%)	3,802 (72.98%)		0.108
	Female		7,795 (59.94%)	2,008 (25.76%)	5,787 (74.24%)		
Age	15-29		2,895 (22.26%)	681 (30.75%)	2,214 (69.25%)		0.000
	30–44		3,753 (28.86%)	933 (24.86%)	2,820 (75.14%)		
	45-59		3,315 (25.49%)	890 (26.86%)	2,425 (73.14%)		
	60-74		2,228 (17.13%)	653 (29.30%)	1,575 (70.70%)		
	75+		814 (6.26%)	259(31.81%)	555 (68.19%)		
Marital status	Married		9,720 (74.74%)	2,451 (25.21%)	7,269 (74.79%)		0.000
	Unmarried		3,285 (25.26%)	965 (29.37%)	2,320 (70.63%)		
Education	Illiterate		3,446 (26.50%)	1,072 (31.10%)	2,374 (68.90%)		0.000
	Primary		3,353 (25.78%)	843 (25.14%)	2,510 (74.86%)		
	Secondary		2,104 (16.18%)	512 (24.33%)	1,592 (75.67%)		
	High school diploma		2,289 (17.60%)	566 (22.90%)	1,723 (77.10%)		
	Higher education		1,813 (13.94%)	423 (23.33%)	1,390 (76.67%)		
Employment status	Employed		3,101 (23.84%)	837 (26.99%)	2,264 (73.01%)		0.033
	Unemployed		1,606 (12.35%)	447 (27.83%)	1,159 (72.17%)		
	Having income without employment		1,438 (11.06%)	404 (28.09%)	1,034 (71.91%)		
	Student		810 (6.23%)	216 (26.66%)	594 (73.34%)		
	Housekeeper		6,050 (46.52%)	1,512 (24.99%)	4,538 (75.01%)		
**Enabling factors**							
Area of residence	Urban		(%)	2,189 (25.16%)	6,509 (74.84%)		0.000
	Rural		(%)	1,227 (28.48%)	3,080 (71.52%)		
Economic status	Q1(Poorest)		2,630 (20.22%)	744 (28.29%)	1,886 (71.71%)		0.000
	Q2		2,583 (19.86%)	748 (28.96%)	1,835 (71.04%)		
	Q3		2,591 (19.92%)	657‌ (25.36%)	1,934 (74.64%)		
	Q4		2,600 (19.99%)	673 (25.88%)	1,927 (74.12%)		
	Q5 (Richest)		2,601 (20.00%)	594 (22.84%)	2,007 (77.16%)		
Basic health insurance	Yes		(%)	3,169 (25.95%)	9,044 (74.05%)		0.000
	No		(%)	247 (31.19%)	545 (68.81%)		
Supplementary health insurance	Yes		12,213 (93.91%)	555 (22.61%)	1,900‌ (77.39%)		0.000
	No		792 (6.09%)	2,861 (27.12%)	7,689 (72.88%)		
**Need factors**							
Number of outpatient healthcare needs	One		10,420 (80.12%)	2,253 (21.62%)	8,167 (78.38%)		0.000
	Two and higher		2,585 (19.88%)	1,163 (44.99%)	1,422 (55.01%)		

**Table 2:** Predictors of self-medication in Iran according to Andersen's Behavioral Model, 2016

		**First model**		**Second model**		**Third model**	
		**OR **	**95% CI**	**OR **	**95% CI**	**OR **	**95% CI**
Sex	Male	0.97	0.84-1.12	0.94	0.82-1.09	1.01	0.87-1.16
	Female	1		1		1	
Age	15-29	1		1		1	
	30–44	1.15	0.99-1.32	1.23	1.06-1.42	1.19	1.02-1.38
	45-59	1.16	0.99-1.36	1.34	1.14-1.58	1.21	1.02-1.43
	60-74	1.12	0.93-1.35	1.31	1.08-1.58	1.20	0.98-1.46
	75+	1.16	0.91-1.46	1.34	1.05-1.70	1.18	0.92-1.51
Marital status	Married	0.79	0.70-0.89	0.79	0.70-0.90	0.80	0.71-0.91
	Unmarried	1		1		1	
Education	Illiterate	1.73	1.44-2.07	1.23	1.01-1.51	1.11	0.90-1.36
	Primary	1.35	1.14-1.59	1.05	0.88-1.26	0.96	0.80-1.16
	Secondary	1.25	1.05-1.48	1.06	0.89-1.27	1.03	0.86-1.23
	High school diploma	1.17	0.98-1.39	1.05	0.88-1.25	1.02	0.85-1.22
	Higher education	1		1		1	
Employment status	Employed	1		1		1	
	Unemployed	0.93	0.80-1.09	0.91	0.78-1.06	0.91	0.78-1.06
	Having income without employment	0.80	0.67-0.95	0.86	0.72-1.03	0.86	0.72-1.03
	Student	0.99	0.78-1.25	1.07	0.84-1.34	1.11	0.87-1.40
	Housekeeper	0.77	0.65-0.91	0.78	0.66-0.93	0.79	0.67-0.93
Area of residence	Urban			1.25	1.13-1.38	1.29	1.17-1.43
	Rural			1		1	
Economic status	Q1(Poorest)			1.21	1.02-1.43	1.14	0.96-1.35
	Q2			1.27	1.08-1.49	1.20	1.02-1.42
	Q3			1.20	1.02-1.40	1.13	0.97-1.33
	Q4			1.17	1.01-1.36	1.13	0.97-1.31
	Q5 (Richest)			1		1	
Basic health insurance	Yes			1			
	No			1.24	1.03-1.48	1.32	1.10-1.58
Supplementary health insurance	Yes			1			
	No			1.22	1.07-1.39	1.18	1.04-1.35
Number of outpatient healthcare needs	One					1	1
	Two and higher					2.96	2.67-3.29

## Conclusion

According to the results, there is a significant rate of self-medication among Iranian households. The rate is higher for single people, older ones, urban residents, lower economic classes, those with no basic and supplementary insurance coverage, and individuals with a greater number of needs for health services. The high prevalence of arbitrary use of Over-The-Counter (OTC) drugs somewhat could be justified by the structure of the country’s health care system that allows access to such drugs. Another point is the high prevalence of arbitrary use of Prescription Only Medicine (POM) medicines which require doctors’ prescriptions. It indicates that the Iranian community is experiencing a health-social problem that needs to be addressed as quickly and effectively as possible. In this regard, more attention must be paid to education on and promotion of drug use culture, because knowledge and awareness are the sources of many human behaviors and practices.
